# Modeling Early Detection and Geographic Variation in Health System Capacity for Alzheimer’s Disease‐Modifying Therapies

**DOI:** 10.1002/alz.089739

**Published:** 2025-01-09

**Authors:** Jodi L. Liu, Lawrence Baker, Annie Chen, Jessie Wang, Federico Girosi

**Affiliations:** ^1^ RAND, Santa Monica, CA USA; ^2^ Pardee RAND Graduate School, Santa Monica, CA USA; ^3^ RAND, Boston, MA USA

## Abstract

**Background:**

The widespread availability of effective disease‐modifying therapies would be a breakthrough in slowing the progression of early Alzheimer’s disease (AD) to later stages of dementia. The purpose of this study is to explore how primary care capacity, variation in patient care‐seeking, and geographic variation in capacity can affect the delivery of AD‐modifying therapies.

**Method:**

We used a county‐level simulation model to assess patient demand and provider supply for the delivery of AD‐modifying therapies. The model simulates the progression of people aged 50 and older through a clinical pathway and through disease states (normal cognitive, mild cognitive impairment, and Alzheimer’s dementia), and compares patient demand to the health care system capacity for detection, diagnosis, and treatment. We developed an interactive, web‐based tool that allows users to adjust key parameters and explore their effects on results.

**Result:**

Care models that enable primary care practitioners to diagnose and evaluate patients for treatment eligibility would make the biggest impact on reducing wait times for dementia specialists and increasing the number of people treated from 2025 through 2044. Improved triage of patients using blood‐based biomarker tests in primary care settings could further reduce caseloads for specialists. The estimated wait times and number of patients treated are sensitive to patient uptake of brief cognitive assessments. There is substantial geographic variation in health care system capacity across the US to detect, diagnose, and treat early AD. Estimated average wait times can be three times longer in rural areas compared to urban areas. States with long wait times include Alaska, Arkansas, Idaho, Mississippi, Montana, Nevada, Oklahoma, and Wyoming.

**Conclusion:**

Widespread delivery of AD‐modifying therapies will require a combination of strategies to communicate the value of detection and treatment to patients, integrate PCPs into the detection and diagnosis pathway, and address capacity disparities across the United States. Further work is needed to evaluate how primary care‐led models of care can widely and effectively evaluate and manage treatment for people with early AD, as well as how technological advancements, such as improved biomarkers and computerized testing, can be integrated into workflows to better serve people living with AD.

